# Night-time immobilization of the distal interphalangeal joint reduces pain and extension deformity in hand osteoarthritis

**DOI:** 10.1093/rheumatology/ket455

**Published:** 2014-02-08

**Authors:** Fiona E. Watt, Donna L. Kennedy, Katharine E. Carlisle, Andrew J. Freidin, Richard M. Szydlo, Lesley Honeyfield, Keshthra Satchithananda, Tonia L. Vincent

**Affiliations:** ^1^Arthritis Research UK Centre for OA Pathogenesis, Kennedy Institute of Rheumatology, Nuffield Department of Orthopaedics, Rheumatology and Musculoskeletal Sciences, University of Oxford, Oxford, ^2^Rheumatology Department, ^3^Therapies Department, Charing Cross Hospital, Imperial College Healthcare NHS Trust, ^4^Centre for Haematology, Hammersmith Hospital, Imperial College London, ^5^Imaging Department, Charing Cross Hospital, Imperial College Healthcare NHS Trust and ^6^Department of Imaging, King’s College Hospital, London, UK.

**Keywords:** osteoarthritis, splint, pain, non-pharmacological therapy, distal, interphalangeal

## Abstract

**Objective.** DIP joint OA is common but has few cost-effective, evidence-based interventions. Pain and deformity [radial or ulnar deviation of the joint or loss of full extension (extension lag)] frequently lead to functional and cosmetic issues. We investigated whether splinting the DIP joint would improve pain, function and deformity.

**Methods.** A prospective, radiologist-blinded, non-randomized, internally controlled trial of custom splinting of the DIP joint was carried out. Twenty-six subjects with painful, deforming DIP joint hand OA gave written, informed consent. One intervention joint and one control joint were nominated. A custom gutter splint was worn nightly for 3 months on the intervention joint, with clinical and radiological assessment at baseline, 3 and 6 months. Differences in the change were compared by the Wilcoxon signed rank test.

**Results.** The median average pain at baseline was similar in the intervention (6/10) and control joints (5/10). Average pain (primary outcome measure) and worst pain in the intervention joint were significantly lower at 3 months compared with baseline (*P* = 0.002, *P* = 0.02). Differences between intervention and control joint average pain reached significance at 6 months (*P* = 0.049). Extension lag deformity was significantly improved in intervention joints at 3 months and in splinted joints compared with matched contralateral joints (*P* = 0.016).

**Conclusion.** Short-term night-time DIP joint splinting is a safe, simple treatment modality that reduces DIP joint pain and improves extension of the digit, and does not appear to give rise to non-compliance, increased stiffness or joint restriction.

**Trial registration:** clinical trials.gov, http://clinicaltrials.gov, NCT01249391.

## Introduction

Hand OA affects 55–70% of the adult population >55 years of age, and DIP joint disease is one of the most common manifestations [1]. Episodes of severe pain or persistent pain and sensitivity to minor knocks are common, contributing to hand dysfunction [2]. Deformity, either radial or ulnar deviation at the joint or loss of full extension (extension lag), is common. Functional deficits and reduced quality of life are well documented in those with DIP joint disease, particularly when associated with other hand joint involvement [2–4]. Aesthetic concerns from hand OA also cause considerable distress and their presence correlates with reduced health-related quality of life [5].

The current burden of symptomatic DIP joint OA partly reflects the lack of effective options, both pharmacological and non-pharmacological, for this disease. Flares and persistent pain at the DIP joint are often poorly responsive to existing analgesics such as paracetamol, oral NSAIDs, or topical NSAIDs or capsaicin. Many clinicians feel steroid injection to the DIP joint is ill-advised, although no controlled studies have been carried out to our knowledge. Ultimately, for very symptomatic joints, DIP joint surgical fusion remains a last resort [6].

Mechanical factors and local inflammation appear important in the initiation of symptoms and progression at any given site in OA. Elegant high-resolution MRI studies in hand OA have shown that collateral ligaments and bony entheses are implicated in DIP joint disease, with inflammatory changes visible in these structures in early and established disease [7, 8]. In acute soft tissue injuries of the DIP joint such as capsular injuries, collateral ligament sprains and tendon avulsion injuries, splinting is routinely used to immobilize healing structures, restoring joint stability and mobility within 12 weeks [9, 10]. Pre-clinical studies by our group suggest that immobilisation of a surgically injured joint abrogates the development of OA [11]. In hand OA there is good evidence from small clinical trials that splinting of the first CMC joint improves pain and function and reduces the need for surgery [12–16]. Anecdotally, we and others have found that splinting of the DIP joint appears to be beneficial in painful interphalangeal OA. We investigated whether custom thermoplastic splinting improves pain, function and deformity of the affected DIP joint and thus might prove a useful therapy for treating OA at this site.

## Methods

### Ethics

Approval was granted by the West London Research Ethics Committee 3 for this clinical trial (REC reference 10/H0706/44). The trial was registered on clinicaltrials.gov (NCT01249391). All participants gave written informed consent to participate prior to screening, according to the Declaration of Helsinki.

### Study design and subjects

**Fig. 1:**
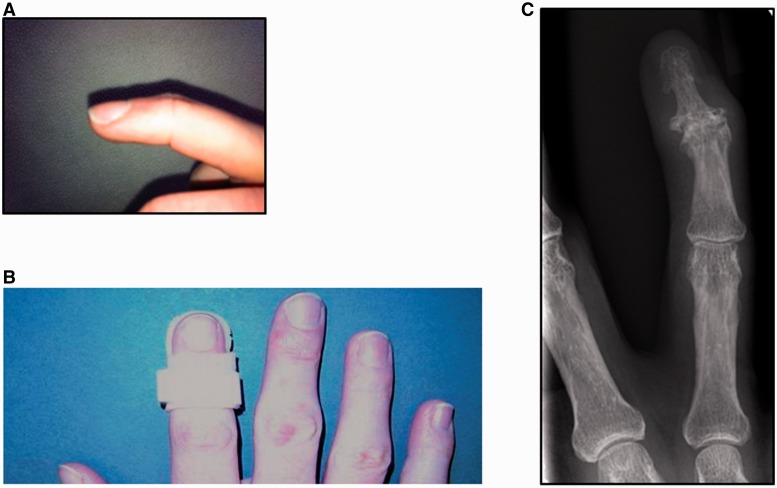
Custom thermoplastic gutter splinting of the DIP joint (**A**) An example of an extension lag deformity of the distal IP joint prior to splinting. There is incomplete extension at the joint on attempted active extension by the individual. (**B**) Splints were fabricated from thermoplastic by a senior hand therapist and adjusted at 6 weeks if necessary to ensure comfort and fit. An example is shown. (**C**) Anteroposterior plain radiograph of a digit from a study subject showing an affected middle finger DIP joint on the right hand. Evidence of radiographic change consistent with OA of the joint is present and there is also radial deviation deformity.

A non-randomized, radiologist-blinded controlled trial was conducted. Participants were patients attending a specialist rheumatology hand clinic at Charing Cross Hospital, Imperial College Healthcare NHS Trust, London, UK. Inclusion criteria were age ≥18 years, a definite diagnosis of hand OA according to ACR criteria [17], evidence of radiographic OA, at least two eligible DIP joints and stable therapies such as NSAIDs for the preceding 4 weeks. Eligible DIP joints for the purposes of this study were defined as those with radiographic OA, pain ≥2 (of 10) on a numerical rating scale (NRS) over the last 7 days and the presence of deformity, defined as at least 7° of radial/ulnar deviation deformity at the joint, with or without loss of full active extension of the joint (extension lag; see Fig. 1A). Exclusion criteria were inflammatory arthritis or other diagnosis contributing to hand pain, a history of psoriasis, planned hand surgery or expected changes to therapy, or IA or systemic corticosteroids in the preceding 3 months, or IA hyaluronans in the preceding 6 months.

### Procedure

During screening, between two and four eligible joints were identified for observation (see above). Of these, an intervention joint was nominated by the patient and clinician to receive a splint. This was a single troublesome, symptomatic joint and could be on either the dominant or non-dominant hand. The other eligible joints were followed as control joints: one of these joints was identified by the clinician (patient and hand therapist blinded) as the nominated control joint, based on specific criteria (in order of preference: similar level of pain as the intervention joint, the same digit on the contralateral hand, an adjacent joint on the contralateral hand, etc.). No placebo was included, as there is no accepted placebo or sham for splinting. A custom gutter thermoplastic splint was made by a senior hand therapist for each subject’s intervention joint at their baseline visit, following assessments (Fig. 1B). Patients were shown how to fit the splint and were asked to wear it every night (for at least 6 and no more than 12 h) for 3 months. Splinting was then discontinued (patients were requested to stop use) and subjects were seen after a further 3 months. A phone call at 2 weeks to ascertain splint comfort and compliance was made, with early review if necessary. Patient-reported adherence to nightly splinting was recorded following questioning at each subsequent study visit. Patients were asked not to alter their pain relief and other hand therapies during the study if possible and any changes were documented at each visit. Anteroposterior radiographs of each of the nominated study digits were acquired prior to initiation of splinting at baseline and the same digits were reimaged at 3 and 6 months (Fig. 1C).

### Outcome measures

The primary outcome measure was average pain in the nominated joints at 3 months. Primary and all secondary outcome measures were recorded at baseline, 3 and 6 months (by the same senior hand therapist or rheumatologist).

#### Pain

Average pain over the preceding week (primary outcome) and worst pain over the preceding week in nominated joints were recorded. The participant was asked to read a standardized question for each and circled a number on an NRS, with anchors of no pain (0) and pain as bad as it can be (10), as recommended in the OARSI guidelines for OA trials [18].

### Disease activity

Tender joint score (1 or 0) for nominated joints was recorded by a rheumatologist. Swelling was measured by joint circumference (in millimetres). Stiffness was recorded in minutes.

#### Functional ability

Pain-free pinch of the nominated joint (in kilograms) was measured with the Biometric E-Link Evaluation System V900S (Biometrics, Gwent, UK). The Biometric E-Link Evaluation System is a calibrated, computerized system incorporating a modified pinch gauge, measuring strength to the tenth of a kilogram (mean of three measurements). The total active range of motion at the DIP joint (degrees) and extension lag (degrees of loss of full extension of the DIP joint on active extension) was measured by goniometer. The range of motion of the PIP and MCP joints of the same digit were also measured. Measurement methods for range, extension, etc. were standardized between therapists and followed American Society of Hand Therapists’ guidelines [19].

#### Joint deformity

The radial or ulnar deviation of each DIP joint was measured by goniometer (degrees). Anteroposterior plain radiograph of each nominated digit was performed at baseline, 3 and 6 months (Fig. 1C). Radiographic deviation (radial or ulnar) was measured by a consultant radiologist blinded to the intervention joint (degrees). All measurements were repeated, blinded to the first reading, at a later date by the same radiologist. All films were also independently measured by another reader (Spearman’s *R* coefficient 0.82).

#### Patient-oriented outcomes

The HAQ, 12-item Short Form (SF-12) and Michigan hand questionnaire (MHQ) were completed at all visits. The MHQ is a self-administered, 37-item questionnaire measuring symptoms and physical function relating to hand and wrist disorders that has been validated in hand OA [20]. The questionnaire was selected because it contains various domains including aesthetic concerns. The score ranges for each hand from 0 to 100, where 0 is maximum disability and 100 is minimum disability. At the end of the study, patients were asked to give a global rating of change regarding whether the intervention finger joint was worse, unchanged or improved compared with the start of the study [21]. They were also asked if they would want to continue the intervention beyond the end of the study.

### Statistical analyses

Power calculation yielded a target sample size of 22 subjects to detect a difference of 2 points on the NRS for average pain with 90% power to detect a statistically significant difference at the 5% level. A change of 1 point on this scale represents the minimum clinically important difference, and a reduction of 2 points represents a feeling of being much improved, which has been employed in other hand OA trials [18, 22]. Changes in the intervention joint and nominated control joint for each subject at 3 and 6 months were calculated by subtracting the baseline score from the scores at either 3 months or 6 months for each digit respectively, and were assessed by the Wilcoxon signed rank test. Differences in change in control and intervention joints at 3 and 6 months (control joint change subtracted from intervention joint change) were assessed by the Wilcoxon signed rank test. *P*-values <0.05 were considered statistically significant. Data analysis was performed using the SPSS version 20 (IBM, Armonk, NY, USA).

## Results

### Study population characteristics

Twenty-six subjects were enrolled in the study. Primary outcome data were available for 23 subjects at 3 months and 22 subjects completed all study visits (2 subjects withdrew after the baseline visit, and there were 2 later withdrawals: 1 patient dropped out for personal reasons (bereavement), 2 because of the need for hand surgery/steroid injection for a non-study joint and only 1 because of intolerance to wearing a splint). Subject characteristics at baseline are shown in Table 1. Their median HAQ, SF-12 and MHQ scores suggested substantial impairment. There was no significant difference in average pain between the nominated intervention and control joints at baseline (*P* = 0.12) (Table 2). Intervention joints were on the dominant hand in about half (12 of 23) of the subjects who completed the 3 month assessment. Of those completing the 3 month assessment, nine patients had a perfect match control (same digit on opposite hand). A further nine had an alternative digit control on the opposite hand. At 2 weeks, one patient required refabrication of their splint to improve fit and comfort. At the 6 week review, a further six patients had minor modifications to improve splint fit. Poor adherence to nightly splinting was reported by only four subjects and this was improved following splint adjustment in all but one subject by 6 weeks (data not shown). Only one patient reported changes in their usual treatment during the intervention period (switch in analgesic). Replication of the analyses without this subject did not affect the results (data not shown).

**Table 1:** Baseline characteristics of the study population

Characteristic	Median (range) or *n* (%)
Age, years	63 (51–78)
Females	23 (88)
Time from diagnosis, years	5.3 (0.3–20)
Body mass index, kg/m^2^	25.8 (18.6–33.5)
Right hand dominant	20 (77)
OA at other (non-hand) sites	12 (46)
Health Activity Questionnaire	1.19 (0–2.3)
SF-12	MCS 42.6 (23.1–61.2) PCS 36.0 (19.4–57.3)
Total Michigan Hand Questionnaire score	47.9 (18.8–85.4)

MCS: mental health component score; PCS: physical health component score.

**Table 2:** Change in primary and secondary outcome measures at 3 and 6 months

Outcome	Baseline value, median (range)		Change in intervention joint from baseline, median (range)				Difference between intervention and control joint, median (range)			
	Intervention	Control	3 month	*P*-value	6 month	*P*-value	3 month	*P*-value	6 month	*P*-value
Average pain (0–10)	6 (1.5, 10)	5 (1.5, 10)	−1.5 (−6, 2)	**0.002**	−2.0 (−8, 4.5)	**0.001**	−0.5 (−7, 3.5)	0.53	−0.5 (−9, 2.5)	**0.049**
Worst pain (0–10)	8.5 (3, 10)	8 (1.5, 10)	−1.0 (−9.5, 4)	**0.02**	−2.5 (−9.5, 1)	**0.003**	0.5 (−9.5, 4.5)	0.83	−0.5 (−11.5, 7.5)	0.40
Joint tenderness (1 = positive, 0 = negative)	1 (0, 1)	1 (0, 1)	0 (−1, 1)	**0.016**	0 (−1, 0)	**0.005**	0 (−2, 1)	0.41	0 (−1, 1)	0.37
Joint circumference, mm	55 (44, 65)	53 (40, 72)	0 (−7, 3)	0.65	0 (−8, 3)	0.75	0 (−2, 10)	0.17	0 (−3, 8)	0.97
Early morning stiffness, min	20 (0, 1440)	20 (0, 1440)	0 (−1440, 1440)	0.89	0 (−1440, 1440)	0.91	0 (−1440, 1440)	0.96	0 (−1440, 30)	0.058
Pinch grip, kg	1.5 (0.2, 4.2)	0.9 (0.2, 3.9)	−0.1 (−0.9, 1.0)	0.34	−0.2 (−1.4, 4.9)	0.57	−0.1 (−1.2, 1.4)	0.37	0.2 (−1.3, 0.8)	0.18
Total range of joint motion, degrees	40 (25, 70)	45 (14, 86)	0 (−10, 35)	0.24	0 (−20, 20)	0.72	3 (−54, 45)	0.85	0 (−56, 61)	0.87
Extension lag of joint, degrees	−15 (−30, 0)	−15 (−35, 4)	2 (−10, 10)	0.096	3 (−10, 10)	**0.039**	5 (−20, 15)	0.075	0 (−24, 30)	0.54
Radiological deviation of the joint, degrees	12 (5, 27)	11 (5, 18)	2 (−7, 8)	0.076	−0.5 (−9, 11)	0.87	0 (−15, 8)	0.93	0 (−21, 17)	0.97
Clinical deviation of the joint, degrees	10 (7, 25)	10 (5, 17)	0 (−25, 6)	0.29	0 (−10, 9)	0.057	−1 (−22, 7)	0.48	0 (−10, 13)	0.56

Values at baseline for primary and secondary outcome measures, their change for the intervention joint at 3 and 6 months (baseline value subtracted from value at *x* months) and the difference in the change between intervention and control joints at 3 and 6 months (the change in value in the control joint subtracted from the change in value in the intervention joint). For all these, a median and range are shown: a negative value represents improvement for all measures except pinch grip, range of motion and extension lag, where a positive value represents improvement. The change in the raw values of the measure in the intervention joint at 3 and 6 months and differences in the change in measures between intervention and control joints were both assessed by Wilcoxon signed rank test. Significant values are shown in bold.

### Sustained reduction in DIP joint pain after splinting

**Fig. 2:**
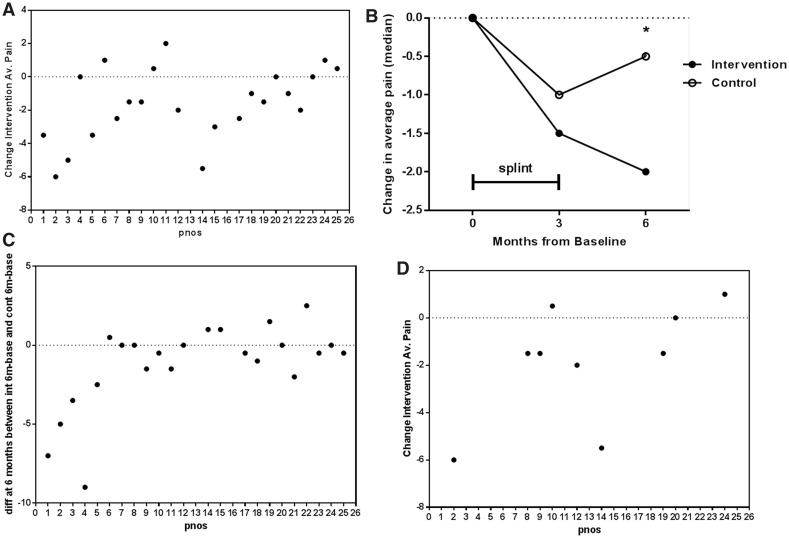
Reduction in pain in DIP joints by splinting For each subject number (pnos), patient-reported pain scores in the intervention joint (int) and control joint (cont) were recorded by a numerical rating scale (0–10). (**A** and **D**) The change in pain scores in the intervention joint at 3 months: the pain at baseline is subtracted from the pain at 3 months. A negative value suggests an improvement in pain. (**A**) The change in average pain in the intervention joint at 3 months (primary outcome) (*P* = 0.002). (**B**) Summary of the median change in average pain from baseline to 3 and 6 months (*P = 0.049). (**C**) The difference in average pain between the intervention and control joints at 6 months is shown for all participants. A negative value suggests more improvement in the intervention joint than the control joint (*P* = 0.049). (**D**) The change in average pain in the intervention joint at 3 months is shown in a predefined subgroup (*n = *9) with a perfect match control joint on the opposite hand (*P* = 0.035).

The average pain reported by subjects over the preceding week (primary outcome measure) was significantly lower in the intervention joint at 3 months compared with baseline (Table 2 and Fig. 2A). This outcome did not appear to be dependent on whether the intervention joint was on the dominant or non-dominant hand (NRS reduced by a median of 1.5 in both groups). The worst pain in the intervention joint in the preceding week was also significantly reduced at 3 months (*P* = 0.02). Ten of 23 (43%) patients had a reduction of ≥2 points on the NRS at 3 months in intervention joint average pain, compared with a similar reduction in only 5 of 23 control joints. When the change in pain in the intervention and control joints was compared at 3 months, differences did not reach significance (Fig. 2B). However, in the 22 patients completing the 6 month visit, the median average pain score in the intervention joint was 2 points lower than baseline and pain was significantly less than in the control joints (*P* = 0.049) (Fig. 2B and C). In a planned subgroup analysis, those who had a contralateral perfect match control digit were assessed. In this group, intervention joint average pain was significantly lower than the control joint at 3 months (*P* = 0.035) (Fig. 2D), supporting the validity of these findings.

### Effect of DIP joint splinting on disease activity and functional ability

Intervention joints were less tender at both 3 and 6 months, although the differences did not reach statistical significance when compared with controls (Table 2). The difference in stiffness of intervention joints and control joints only approached significance at 6 months (*P* = 0.058) (Table 2). No difference was seen at 3 or 6 months in joint circumference, pinch grip strength or total range of motion of the DIP joint (Table 2). There was also no difference in HAQ or SF-12 scores over the course of the study period. Of the 18 patients who had intervention and control joints on opposite hands, there was no difference between MHQ scores for intervention and control hands. There was no evidence of reduced range of motion in the splinted joint or adjacent PIP or MCP joints, which was a theoretical concern when immobilising a joint, even on a nightly basis (data not shown).

### Sustained improvement in extension lag deformity by DIP joint splinting

In all but 2 of 26 subjects at baseline, the radial or ulnar deviation deformity was passively correctable by at least 5° (median correction 10°, range 3–15°), suggesting that splinting might achieve correction. The change in joint deviation on plain radiograph (radiologist blinded to the intervention digit) approached significance at 3 months (*P* = 0.076). Similarly, clinical measurement of resting joint deviation showed a trend towards improvement at 6 months, but did not reach significance (*P* = 0.057). It should be noted that clinical and radiological measures of deviation only correlated moderately with each other (Pearson *R* = 0.63, *P* < 0.0001).

**Fig. 3:**
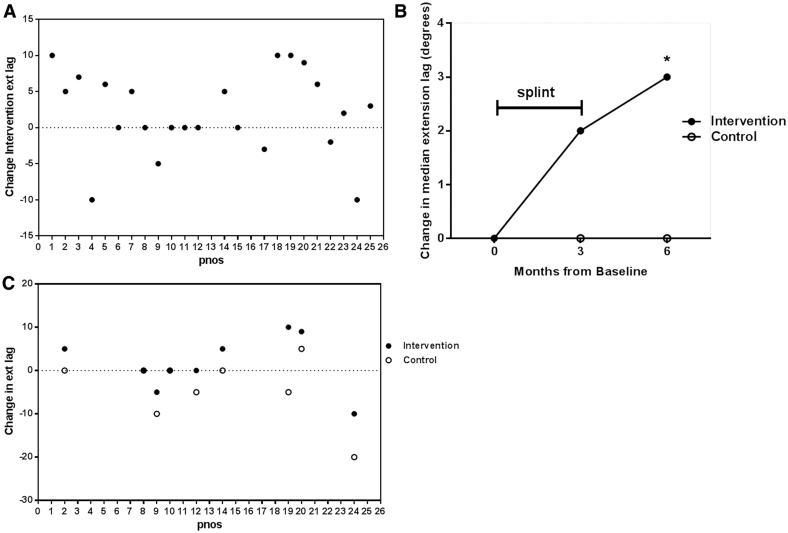
Improvement in extension lag deformity in DIP joints by splinting For each subject number (pnos), the degrees of incomplete extension on attempted active extension (ext lag) were recorded for the intervention joint and control joint. A positive value suggests an improvement in deformity and a negative value suggests a deterioration. (**A**) The change in extension lag deformity in the intervention joint at 3 months. The lag at baseline is subtracted from the lag at 3 months (*P* = 0.096). (**B**) Summary of median change in extension lag deformity from baseline to 3 and 6 months. *P = 0.039 in intervention joints only. (**C**) The change in extension lag deformity at 3 months in control joints (clear circles) and in intervention joints (black circles) in a predefined subgroup with a perfect match control on the opposite hand (*n = *9; *P* = 0.016).

Extension lag deformity (a flexion deformity at the DIP joint) occurred frequently, with all except one patient having at least 8° at baseline. The change in intervention joint extension lag at 3 months compared with baseline is shown in Fig. 3A, and was significantly improved by 6 months (*P* = 0.039) (Fig. 3B). This change did not reach significance when compared with controls for the whole study population (*P* = 0.075). However, in the planned subgroup analysis comparing intervention joints with same-joint controls on the contralateral hand, extension lag was significantly improved in intervention joints at 3 months (*P* = 0.016) (Fig. 3C).

### Changes perceived by patients

Of the 23 patients who completed the study, 17 (74%) reported overall improvement following the intervention and 14 (61%) wanted to continue use of the splint beyond the study. Importantly, there was no reported increase in stiffness in the DIP joint or adjacent joints on the same digit. *Post hoc* testing of aesthetic domains for the MHQ for the 18 subjects who had intervention and control joints on opposite hands did not show any change over the trial period (data not shown).

## Discussion

To our knowledge this is the first trial of custom splinting in IP joint OA or any form of chronic IP joint arthritis. Our results suggest that short-term night-time DIP joint splinting is a simple treatment modality that reduces DIP joint pain and also appears to improve extension deformity. Interestingly, the effects were sustained, and even increased beyond the use of the orthotic, suggesting that such devices may have symptom- and disease-modifying properties. Splinting does not, if carried out nightly for a restricted period of time, seem to give rise to non-compliance, increased stiffness or restriction of range of motion. Splinting for 24 h may have brought about a greater change in outcomes, but is less likely to have been well tolerated and may have led to a stiffer joint. The study does not answer how long nightly splinting can safely be continued, or whether there is an optimum length of time for splinting. Anecdotally, several of our patients continue to use these DIP joint splints on an intermittent basis with ongoing benefit. Ikeda *et al.* [23] reported a case series of patients where a prefabricated plastic sleeve led to improvement in reported pain in hand OA. Our trial differs in several important respects: the study population had deforming disease; custom, adjustable splinting was employed; and control joints were monitored in the study, as a means of internal control. The reasons for splinting ameliorating pain are not well understood, but are thought to result in part from joint protection from ongoing aggravating mechanical factors, with resulting reduction in inflammation or promotion of tissue healing, as seen in joint distraction [24].

Design of non-pharmacological studies in hand OA is challenging. The lack of a suitable placebo intervention to which investigator and patient are blinded is a recognized issue in this type of study. Devices such as tubi-grip or digi-sleeves were considered but may have acted to immobilize and increase joint awareness and thus were not felt to be appropriate control or sham arms. This was a pragmatic trial and, as such, we chose to treat a single troublesome, symptomatic joint rather than identify a joint by randomization. However, identifying a similar control joint in all patients proved to be challenging. Hand OA is often thought of as a symmetrical disease, but as our subanalysis demonstrates, only nine patients had eligible contralateral same-joint controls, and for some subjects there was a larger-than-ideal difference in levels of pain and other secondary measures between intervention and nominated control joints at baseline (although no difference overall between groups). It is possible that the trend towards worse pain in the selected intervention joints could have introduced a bias, favouring response. However, it is difficult to be sure that a less painful control joint would necessarily be less responsive to change than a more painful joint: there is little evidence to support this. Large changes over the study period were observed in control joints for some measures, highlighting the natural history of this disease. Such differences may represent different stages of disease in different joints, which could make an intervention effect harder to detect. The fact that the controls were within-participant and simultaneously monitored meant that they were not independent of the intervention joint. This is arguably a strength of the study and intentionally attempts to control for disease fluctuations within the individual over time. The trial was powered to detect a difference in average pain over a 3 month period and it is likely that a far larger population would be required to fully explore the effects of splinting on some of the other selected secondary outcomes. The lack of change in global measures such as the SF-12, HAQ and MHQ was perhaps not surprising where only a single joint was treated and other affected hand joints were present. Validated responsive measures for single joint assessment would be of value for future clinical trials of this nature.

These participants were selected because of established, often advanced hand OA. Given our findings, it would be of interest to assess splinting in early hand OA, at the time of initial pain, soft tissue change and early/correctable deformity. This would arguably be at a more modifiable point in the disease. DIP joints were selected for this current study for a number of reasons. PIP joint OA is reported to give rise to even greater functional deficits and symptoms can be difficult to manage, and splinting may be less well tolerated at this site [2].

The study demonstrates that small clinical trials can be effective in demonstrating clinically important effects. A larger trial would have required a multicentre approach, which might be challenging because of the bespoke nature of the orthotic. This would be necessary if more restrictive criteria or subgroup analysis were to be attempted. An alternative would be a prefabricated splint, available in different sizes. However, in our experience, good comfort and fit is unlikely to be achieved using a prefabricated rigid splint in anything other than very early disease, given the inherent joint deformity, and this would lead to poor compliance. Some of the benefits in this study may have arisen because of the ability to adjust individual splints during the splinting period as joint correction occurred.

### Conclusion

Custom thermoplastic splinting of the DIP joint in painful, deforming hand OA improves pain and, to a lesser extent, extension lag deformity. These findings would justify the further assessment of night-time splinting in larger studies as a routine therapeutic option for both distal and PIP joint OA.

**Rheumatology key messages**

- Night-time thermoplastic splinting of osteoarthritic DIP joints improves pain and extension lag deformity.
- IP joint splinting is a well-tolerated, novel, joint-specific treatment for hand OA and should be explored in larger clinical trials.
